# Comparison of Analgesic Effect between Gabapentin and Diclofenac on Post-Operative Pain in Patients Undergoing Tonsillectomy

**DOI:** 10.5812/atr.10441

**Published:** 2013-12-01

**Authors:** Mario I Ortiz, Luis C Romero-Quezada

**Affiliations:** 1 Medicine Academic Area, Health Sciences Institute at the Autonomous University of Hidalgo State, Pachuca, Hidalgo, Mexico

**Keywords:** Gabapentin, Diclofenac

Dear Editor,

We have examined attentively the latest manuscript by Yeganeh Mogadam et al. entitled “Comparison of Analgesic Effect between Gabapentin and Diclofenac on Post-Operative Pain in Patients Undergoing Tonsillectomy” available in Archives of Trauma Research (1). Ninety patients were included to receive 20 mg/kg oral gabapentin (n = 30), 1.0 mg/kg rectal diclofenac (n = 30) or placebo (n = 30) preoperatively. Pain was evaluated postoperatively at 2, 6, 12 and 24 hours. Authors concluded that gabapentin and diclofenac reduced postoperative pain and opioid consumption without any obvious side effects.

Diclofenac is highly bound to serum proteins (≥ 99.5%) and has a relatively low volume of distribution (0.12 to 0.17 L/kg) (2, 3). Diclofenac easily penetrates the synovial fluid and crosses the placenta (2,3). However, diclofenac does not easily cross the blood-brain barrier. It has been proved that the diclofenac concentrations in cerebrospinal fluid is 8.22% compared to its value in plasma (4). There are reports wherein the anti-inflammatory and antinociceptive effect of diclofenac cannot be directly explained by circulating concentrations in humans (2, 3). Therefore, it has been suggested that diclofenac effects are mainly mediated via a local action in target tissues. In the present study, it is probable that diclofenac produced prostaglandins inhibition to oropharynx level and with that decreasing the sensitization of nociceptors at this level. On the other hand, although gabapentin is a structural analogue of GABA, which does not cross the blood-brain barrier, gabapentin penetrates into the central nervous system (5). Gabapentin binds to plasma proteins and it is not metabolized by nor inhibits hepatic enzymes that are responsible for the metabolism of other drugs (6). It is well known that gabapentin has analgesic and antihyperalgesic effects and is used in the control of clinical pain (6, 7). In this sense, the properties of gabapentin have been attributed to an action on the central and peripheral nervous system. Therefore, it is suggested that the gabapentin-induced analgesic effect in the present study was mediated by pain modulation at local level and on the descending pathways. In the present study, children and adult patients were evaluated together. It has been suggested that children may need moderately higher dosages of gabapentin to reach plasma concentrations comparable with those found in adults (8). Likewise, differences have been found in the volume of distribution, Cmax and clearance of diclofenac between children and adults (9, 10). Therefore, all the participants should have been evaluated separately to obtain a more consistent result. 

In the case of the pain intensity, authors stated on the background section that the post-operative pain in patients undergoing tonsillectomy may be as high as 70 in the scale of visual analog scale. However, the pain intensity in the present study was very low (from 1.53 to 3.43). Therefore, the effects of gabapentin and diclofenac appear very scarce or nil. On the other hand, the comparison of pain intensity in the three groups in the manuscript seems wrong. Whereas authors state that the patients’ pain in gabapentin group was significantly less than that of the placebo group (P < 0.05) and the mean pain intensity in diclofenac group at 6 hours after the surgery was significantly less than that of the placebo group (P < 0.05). Data shows that the pain intensities at 6, 12 and 24 hours in the gabapentin and diclofenac groups were bigger than in the placebo group. Only the pain intensities at 2 hours in the treated groups were lower than in the placebo group. This mistake was probably a measurement mistake, made by the authors.
